# Experimental Verification of Inferred Regulatory Interactions of *EARLY FLOWERING 3* ( *GmELF3-1* ) in *Glycine max*

**DOI:** 10.17912/micropub.biology.000687

**Published:** 2022-11-22

**Authors:** Michelle Alcantara, Patrick Acosta, Ara Azatian, Carlos Calderon, Kevin Candray, Natalie Castillo, Luis Coria-Gomez, Jose Duran, Justina Fam, Diego Hernandez-Segura, Lennix Hidalgo, Carlos Huerta, Shane Jordan, Kimberly Kagan, Karla Loya, Eduardo Martinez, Kirill Musaev, Roxana Navarro, Narek Nazarians, Robert Paglia, Gabriela Robles, Taylor Simmons, Shawn Smith, Faisel Soudani, Emily Valenzuela, Jessica Villalobos, Hira Iftikhar, Yoshie Hanzawa

**Affiliations:** 1 Department of Biology, California State University Northridge; 2 Department of Biology, BIOL 481 Plant Physiology, California State University Northridge

## Abstract

Understanding the roles of evening complex (EC) genes in the circadian clock of plants can inform how diurnal transcriptional loops in the clock gene network function to regulate key physiological and developmental events, including flowering transition. Gene regulatory interactions among soybean’s circadian clock and flowering genes were inferred using time-series RNA-seq data and the network inference algorithmic package CausNet. In this study, we seek to clarify the inferred regulatory interactions of the EC gene *GmELF3-1.* A gene expression analysis using soybean protoplasts as a transient model indicated regulatory roles of *GmELF3-1* in expression of selected flowering genes.

**Figure 1. Expression of GFP - GmELF3-1 and inferred downstream genes in GFP - GmELF3-1 transfected protoplasts.**
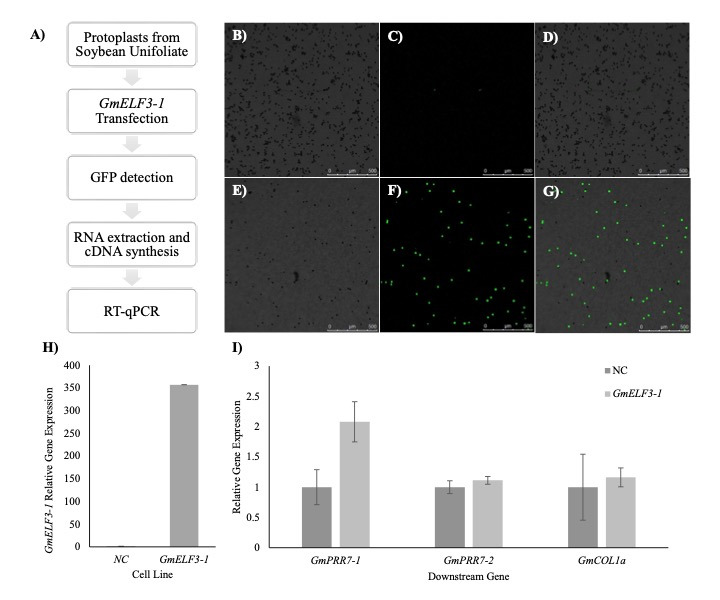
**(A)** Methodological diagram for this study. Protoplasts were used as a transient model for examining the regulatory roles of *GmELF3-1* in inferred downstream genes. **(B-G)** Representative images showing GFP expression in the non-transfected negative control protoplasts (B-D) and in *GFP-GmELF3-1* transfected protoplasts (E-G) under fluorescent microscopy at Zeitgeber time (ZT) 12:00. B and E: bright field, D and F: GFP, and D and G: merged images. **(H)** Relative expression of *GmELF3-1* in negative control (NC) and *GFP-GmELF3-1* transfected protoplasts ( *GmELF3-1* ) at ZT12:00 by RT-qPCR in two biological samples with three technical replications. **(I)** Relative expression of inferred downstream genes: *GmPRR7-1, GmPRR7-2,* and *GmCOL1a* at ZT12:00, in negative control (NC) and *GFP-GmELF3-1* transfected protoplasts ( *GmELF3-1* ) by RT-qPCR in two biological samples with three technical replications. Relative expression was calculated using the housekeeping gene *GmPBB2* as described in Wu *et al.* 2014 and Livak and Schmittgen 2021.

## Description

The circadian clock gene network in plants is a transcriptional landscape in which a panoply of regulatory genes controls each other during a 24-hour diurnal period (Harmon et al. 2018; Creux and Harmer, 2019; Roland and Davis 2019). Each of these genes is expressed rhythmically in alternating patterns of upregulation and repression in the 24-hour period, creating the circadian oscillator. Thus, the circadian clock genes function as molecular timekeepers that influence many key physiological processes of plants. These include the expression of genes related to hormone signaling, shoot development, floral growth, and response to biotic and abiotic stressors in the environment (Sanchez et al. 2011; Greenham and McClung 2015).

Recent studies suggest that a set of circadian oscillator genes expressed in the evening phase, known as the evening complex (EC) that consists of *LUX ARRHYTHMO* ( *LUX* ), *EARLY FLOWERING 3* ( *ELF3* ), and *EARLY FLOWERING 4* ( *ELF4* ) genes, plays important roles in environmental responses of plants (Creux and Harmer, 2019). Mutations in the *ELF3* gene in *Arabidopsis* have promoted earlier flowering transitions than wild type in short day conditions (Hicks et al., 2001). Further studies have indicated that mutations in any of the EC genes also result in early flowering transitions (Doyle *et al* ., 2002). Genetic variation in the EC genes has been shown as the main cause of variation in photoperiod sensitivity and flowering time in multiple crop species (Liew *et. al.,* 2009; Gawroński *et al.,* 2014; Li *et. al.,* 2016; Liew *et. al* ., 2014). The roles of *ELF3* in light-signaling, temperature-sensing and photoperiodic flowering, also indicate its strong ties to the domestication of plants (Faure *et al.,* 2012; Weller *et. al.,* 2012; Zakhrabekova *et. al.,* 2012).

The *ELF3* gene has been identified as the causal gene of the *J* locus which accounts for flowering time variation in soybean (Lu *et al.* , 2017). A loss-of-function allele of the *J* locus causes late flowering in short days due to the inhibition of the *E1* gene that represses the flowering promoter *FT* genes. The *J* locus is thought to have allowed soybean cultivation expansion to equatorial regions by extending the vegetative phase development in short days. The diurnal expression pattern of *GmELF3* was abolished in plants carrying the loss-of-function *j* alelle, indicating that an intact *GmELF3* is essential to maintain its own expression pattern. However, the effect of *GmELF3* on the soybean circadian rhythm is yet to be characterized.

This study used soybean protoplast cells as a transient model to express the EC gene *GmELF3-1* and examine the regulatory interactions by RT-qPCR. The objective of this study is to gain a better understanding of the role of *GmELF3-1* in the circadian clock of the short-day plant *Glycine max* by elucidating what genes *GmELF3-1* may control and how *GmELF3-1* regulates expression of these downstream genes.

Regulatory interactions among 82 soybean’s circadian clock and flowering genes were inferred with the in-house time-series RNA-seq data and the network inference algorithmic package CausNet (Wu *et al.* 2019). A complete set of soybean homologs of the core clock oscillator genes in *Arabidopsis* : *LATE ELONGATED HYPOCOTYL* ( *LHY* ), *CIRCADIAN CLOCK ASSOCIATED 1* ( *CCA1* ), *PSEUDO RESPONSE REGULATOR* ( *PRR* ) genes, *TIMING OF CAB EXPRESSION 1* ( *TOC1* ), *ELF3* , *ELF4* , *LUX* and *GIGANTEA* ( *GI* ), was included in addition to the 74 genes that were previously used in Wu *et al.* 2019. Cytoscape provided a visual aid for observation of inferred regulatory interactions with upregulation or downregulation of regulatee genes by regulator genes in differing temperature and photoperiod conditions. Respective weights in each inferred interaction determined highly reliable regulatory interactions.

Based on the Cytoscape observation, we identified candidate downstream genes of *GmELF3-1* that had relatively strong reliability weights. *GmPRR7-1* and *GmPRR7-2* were predicted to be downregulated by *GmELF3-1* with a confidence weight of 0.62 and 0.72, respectively, in the photothermal condition long day at 25 ^o^ C. The *CONSTANS-LIKE 1a* ( *GmCOL1a* ) gene was predicted to be upregulated by *GmELF3-1* with a confidence weight of 1.0 in long day at 16 ^o^ C. In an attempt to experimentally verify the inferred regulatory interactions of *GmELF3-1* and its respective downstream genes, soybean protoplasts were isolated from unifoliate leaves and transfected with the *GFP-GmELF3-1* plasmid DNA (Table 2) *.* Transfected protoplasts were harvested after an overnight incubation at the Zeitgeber time point ZT12:00 and flash frozen with liquid nitrogen. RNA was extracted and cDNA libraries prepared, and RT-qPCR was carried out for gene expression analysis. The *GFP-GmELF3-1* transfected protoplasts showed GFP fluorescence at ZT12:00, while the negative control protoplasts showed no fluorescence (Figure 1B-G). Approximately 85% of the *GFP-GmELF3-1* transfected protoplasts had the GFP signals, confirming that transfection was successful. Upregulation of *GmELF3-1* expression was observed in the *GFP-GmELF3-1* transfected protoplasts with a 350-fold increase compared with the negative control, indicating that *GmELF3-* 1 was highly expressed (Figure 1H). Expression of an inferred downstream gene, *GmPRR7-1* , showed a twofold increase compared to the negative control, whereas there was no significant difference in expression levels of *GmPRR7-2* and *GmCOL1a* between the *GFP-GmELF3-1* transfected protoplasts and the negative control (Figure 1I) *.*

Our expression study indicates that *GmELF3-1* upregulates *GmPRR7-1* at ZT12:00 under non-flowering inductive long day at 25 ^o^ C, suggesting a regulatory interaction between *GmELF3-1* and *GmPRR7-1* . Limitations of this study include our sampling scheme targeting only a single time point. Circadian clock genes and many flowering genes exhibit diurnal expression patterns with distinct peak expression time. For example, published findings have shown that *GmCOL1a* has peak expression at the end of the night (Wu *et al.* 2014). Further clarification of the inferred regulatory interactions in our future study will require more time points in a 24-hour period to better capture changes in rhythmic gene expression. In addition, although our expressional study showed the regulatory effect of *GmELF3-1* in *GmPRR7-1* expression, it cannot exclude that an indirect regulation through a feedback loop within the circadian clock network may have caused this effect, as the circadian clock in *Arabidopsis* is well known to consist of multiple feedback loops involving *PRR* genes. Nevertheless, this study optimizes strategies for inference and experimental verification of gene regulatory networks and provides further evidence of regulatory roles of the *GmELF3-1* gene.

## Methods

**Plant growth conditions** *Glycine max* Williams 82 cultivar seeds were grown in Sunshine Mix #4 Professional Growing mix with Mycorrhizae and Vermiculite in a 6:1 ratio respectively. The plants were then placed in a growth chamber with 14-hour light exposure per day at 25 °C. The pH and moisture conditions of the soil in each pot were regularly monitored, ensuring the soil moisture range is within 40%-50% and the pH remains at around 7.

**Protoplast isolation** Fully expanded unifoliate leaves of soybean seedlings at 6-8 days after germination were used for protoplast isolation following the procedures described in Wu and Hanzawa, 2018 with modifications. An enzymatic digestion solution consisting of 0.02M of MES pH 5.7, 1.50% w/v Cellulase (R10), 0.50% w/v Macerozyme, 0.20% w/v Pectolyase Y-23, 0.4M D-Mannitol, and ddH2O was added to 90% of the final volume and left in a water bath set to 55 °C for 10 minutes. 0.1M CaCl2, 0.75% v/v of BSA, and remaining ddH2O was added to its final volume at ambient temperature and filtered by a 0.45-μ syringe filter. The primary vein and bottom epidermal layer of a soybean unifolicate was removed using a leaf-tape sandwich method following the procedures for *Arabidopsis* (Wu *et al* . 2009) with modifications. The mesophyll cells were incubated for 2.5-3 hours at 22 °C in low light, with gentle agitation of 100 rpm and observed every 30 minutes under the microscope. W5 solution (154mM NaCl, 125 mM CaCl2, 5 mM KCl, 2mM MES pH 5.7) was added and filtered through a 70-μm nylon mesh. The concentration of the protoplasts was identified with a hemocytometer and suspended in prechilled W5 solution to a final concentration of 2 x 10 ^5^ mL ^-1^ . The protoplasts were incubated on ice for 30 minutes, then centrifuged at 100 x g for 5 minutes and the pellet was resuspended in MMg solution (4 mM MES pH 5.7, 400 mM D-Mannitol, 15 mM MgCl2) to a final concentration of 2 x 10 ^5^ mL ^-1^ .

**Protoplast transfection** Protoplast transfection was carried out following the procedures described in Wu and Hanzawa, 2018 *.* To 2 mL aliquots of protoplasts, 10 μg of plasmid DNA were added. To each sample, PEG solution (20% w/v PEG4000, 400 mM D-Mannitol, 100 mM CaCl2) was added and incubated for 12 minutes. W5 solution was added to cease transformation and was centrifuged at 100 x g for 5 minutes. 2 mL of WI solution (4 mM MES pH 5.7, 400 mM Mannitol, 15 mM MgCl2) resuspended the pellet. To a 6 well tissue culture plate, 1 mL of 50% v/v sterile calf serum was added to the wells and 1 mL of the transfected protoplasts were placed into the culture plate. The plate was covered and incubated at 22 °C overnight in the dark. The following day, the viability and GFP expression of the protoplasts were examined using fluorescent microscopy before proceeding to RNA extraction.

**Plasmid construction**

The full length *GmELF3-1* cDNA (https://phytozome-next.jgi.doe.gov/report/gene/Gmax_Wm82_a2_v1/Glyma.04G050200) was amplified, cloned into the pCR8 vector (Invitrogen) and sequenced for verification. The *GmELF3-1* cDNA was transferred to the p2FGW7 vector (VIB-UGENT Center for Plant Systems Biology; https://gatewayvectors.vib.be/collection/p2fgw7 ) via the LR Gateway recombination following manufacturer’s instructions (Invitrogen). The p2FGW7-ELF3-1 plasmid and further information about the plasmid are available upon request by contacting the corresponding author Yoshie Hanzawa (yoshie.hanzawa@csun.edu).

**RNA isolation**

Protoplast cells were washed with 1X PBS prior to disruption by Lysis/Ethanol Solution provided by the Invitrogen™ RNA Kit. Samples were centrifuged at maximum speed per the kit’s protocol. The supernatant was added to a Micro Filter Cartridge Assembly and centrifuged to latch the RNA in the filter. The filter was then washed with Wash Solution 1 and Wash Solution 2/3 sequentially. Preheated ddH _2_ O was used for eluant. The eluant was followed by a DNase treatment, provided by the Invitrogen™ RNA Kit. The concentration was measured using NanoDrop Spectrophotometer.

**Quantitative RT-PCR**

cDNA was synthesized from the respective RNA extraction using the Bio-Rad™ iScript cDNA synthesis kit. cDNA concentration was kept to 1000ng/uL and diluted to 5ng/uL for RT-qPCR application. Applied Biosystems 7300 Real-Time PCR System was used for the instrument with Applied Biosystems Power SYBR Green PCR Master Mix. Thermal Cycler settings had the first stage at 50 ^o^ C for 2 minutes for 1 cycle, second stage at 95 ^o^ C for 10 minutes with 1 cycle, and the third stage at 95 ^o^ C for 15 seconds for 40 cycles. Last stage was 60 ^o^ C for 1 minute. The housekeeping gene *GmPBB2* was used to standardize gene expression and relative expression was calculated as described in Wu *et al.* 2014 and Livak and Schmittgen 2001.

## Reagents

**Table:** 

**Strain**	**ID**	**Available From**
Williams 82	PI 518671	USDA

**Table 1. Strain of *Glycine max* used for protoplast isolation.**

**Table:** 

**Plasmid**	**Gene ID**
p2FGW7-ELF3-1	Glyma.04G050200

**Table 2: Plasmid name with vector backbone and its respective Gene ID.**

**Table:** 

**Name**	**Primer sequence (5’-3’)**	**Target Gene Name**	**Gene ID**
GmPBB2-F GmPBB2-R	TGCCGAAGAAACGCAATGCTTCAA TGCAGCAAGTGAACCTGATCCCAT	*GmPBB2*	Glyma.14G01850
GmELF3-1-F GmELF3-1-R	TGTTCTGCCACTCAACCCAA TGATTGGCGTGAGTTACATT	*GmELF3-1*	Glyma.04G050200
GmPRR71-F GmPRR71-R	TATGAAGTTATTGAAGCAGC AGAATCATGAGATGACATCA	*GmPRR7-1*	Glyma.10G048100
GmPRR72-F GmPRR72-R	GTCTGCTTTCTCAAGGTACA GGAGGATTGCCGCTAGAATG	*GmPRR7-2*	Glyma.13G135900
GmCOL1a-F GmCOL1a-R	CGCCTCGCTGACGTGGCACG TTGTCGTTGTTGCCGGGGGC	*GmCOL1a*	Glyma.08G255200

**Table 3. Primers used for RT-qPCR.** Primers were designed based on the CDS sequences of target genes in *Glycine max Wm82.a2.v1* .
